# Comparison of Mortality and Morbidity of Robotic Versus Laparoscopic Radical Nephrectomy for the Treatment of Renal Cell Carcinoma—An Analysis of the National Surgery Quality Improvement Program (NSQIP) Targeted Nephrectomy Database

**DOI:** 10.3390/curroncol32060358

**Published:** 2025-06-17

**Authors:** Vatsala Mundra, Siqi Hu, Renil Sinu Titus, Eusebio Luna-Velazquez, Zachary Melchiode, Jiaqiong Xu, Carlos Riveros, Sanjana Ranganathan, Emily Huang, Brian J. Miles, Dharam Kaushik, Christopher J. D. Wallis, Raj Satkunasivam

**Affiliations:** 1 Department of Urology, Houston Methodist Hospital, Houston, TX 77030, USA; vmundra@houstonmethodist.org (V.M.); zmelch@lsuhsc.edu (Z.M.); sranganthan@houstonmethodist.org (S.R.); dkaushik@houstonmethodist.org (D.K.); 2 Center for Health Data Science and Analytics, Houston Methodist Research Institute, Houston, TX 77030, USA; 3 Division of Urology and Surgical Oncology, Department of Surgery, Princess Margaret Cancer Centre, University Health Network, University of Toronto, Toronto, ON M5S 1A1, Canada; christopher.wallis@sinaihealth.ca; 4 Division of Urology, University of Toronto, Toronto, ON M5S 1A1, Canada; 5 Division of Urology, Mount Sinai Hospital, Toronto, ON M5G 1X5, Canada

**Keywords:** renal cell carcinoma, radical nephrectomy, laparoscopic surgery, robot-assisted surgery

## Abstract

Objectives: To compare the perioperative complications between robot-assisted (RARN) and laparoscopic (LRN) radical nephrectomy for the treatment of renal cell carcinoma (RCC). Methods: We conducted a retrospective study using the National Surgical Quality Improvement Program (NSQIP) Nephrectomy-Targeted database from 2019 to 2021. After using propensity score matching, we assessed the association between LRN vs. RARN and the outcomes of interest (primary outcomes of 30-day mortality, return to the operating room, myocardial infarction, and stroke; and secondary outcomes of perioperative complications and nephrectomy-specific outcomes). Results: Among the 1545 patients in the study (mean age: 62.9 ± 11.8 years), 722 underwent RARN and 823 underwent LRN. We did not observe any differences in the major complications between the two approaches. However, LRN was associated with an increased chance of surgical site infections compared with RARN (LRN 2.68% vs. RARN 1.19%, *p* = 0.047). LRN was also associated with a higher likelihood of a prolonged length of stay (OR 1.54, 95% CI: 1.15, 2.06, *p* = 0.004) and had a 2.7 times higher chance of conversion rate to open surgery (OR 3.70, 95% CI: 3.25, 4.15, *p* < 0.001) relative to RARN. However, RARN was associated with a longer operative time than LRN (estimated coefficient 30.67, *p* < 0.001). Conclusion: We found no significant difference in the major complications between RARN and LRN for patients undergoing radical nephrectomy. At the expense of a somewhat longer operative time, RARN was associated with a lower risk of SSI and a lower conversion rate to open RN. LRN and RARN should both be considered and selected on an individualized basis using tumor, patient, and physician factors.

## Simple Summary

In patients with kidney cancer that exceeds 4 cm, radical nephrectomy is the mainstay of treatment. In our large-scale, multi-institutional study, we compared robotic-assisted radical nephrectomy vs. laparoscopic radical nephrectomy in the contemporary setting. We observed that although there were no differences in major complications (death, readmission, heart attack, or stroke), laparoscopic surgery was associated with an increased risk of surgical site infection, a prolonged length of stay, and an increased conversion rate to open surgery. We also found that robotic surgery is associated with an increased time in the operating room. However, while robotic-assisted surgery is associated with decreased complications, previous studies have shown that robotic-assisted surgery requires more monetary resources than laparoscopic. Overall, when choosing between conducting a radical nephrectomy in a robot-assisted manner or laparoscopically, surgeons should consider specific patient factors, the risk of perioperative complications, and incorporate value-based care.

## 1. Introduction

Most patients diagnosed with renal cell carcinoma (RCC) are found to have localized disease (90%) [1]. Surgery remains the mainstay of treatment for many patients, with radical nephrectomy (RN) being the reference standard for managing larger tumors > 4 cm [2]. Minimally invasive approaches such as laparoscopic radical nephrectomy (LRN) and the robot-assisted iteration (robot-assisted radical nephrectomy (RARN)) have been increasingly utilized [3,4]. Compared with traditional LRN, RARN offers potential advantages such as improved surgical dexterity and enhanced anatomical visualization during surgery, which can improve dissection, tumor identification, and the control of renal vessels, all critical for reconstructive surgery, specifically partial nephrectomy [5,6]. It is unclear whether these potential benefits would translate to improved postoperative outcomes for patients undergoing RN. Although RARN is chosen for inherently larger tumors, and for patients with more comorbidities, previous studies have found no significant differences in the occurrence of major surgical complications with LRN and RARN [2,7,8,9,10,11,12,13,14]. However, RARN is associated with higher overall costs [9,10,15].

Therefore, using the American College of Surgeons-National Surgical Quality Improvement Program (ACS-NSQIP) Nephrectomy-Targeted databases, we sought to comprehensively compare the surgical complications of LRN vs. RARN between 2019 and 2021.

## 2. Methods

### 2.1. Study Design and Study Population

We used the ACS-NSQIP Targeted Nephrectomy database, which began in 2019 and offers more detailed information compared with the general NSQIP dataset about specific surgical procedures, including details of robotic-assisted approaches. The database was used to identify patients with kidney cancer who underwent either LRN or RARN from 2019 to 2021.

NSQIP includes 30-day surgical outcomes for over 700 North American hospitals, representing approximately 20% of U.S. hospitals across various surgical subspecialties. The database includes demographic information, previous medical history, as well as preoperative, intraoperative data, and 30-day postoperative outcomes. The detailed data sampling procedures of ACS-NSQIP have been previously described in detail [16].

### 2.2. Inclusion and Exclusion Criteria

Using the International Classification of Diseases for Oncology code C64, we identified patients who underwent RN for RCC. Our study excluded non-elective cases, patients aged 90 or older, individuals with preoperative renal failure or metastatic disease, those with an American Society of Anesthesiologists (ASA) score of 5, patients with preoperative open/infected wounds, as well as those who had any complications prior to the time of surgery. We excluded cases with open, endoscopic, hybrid, unknown, other, and any other unclear minimally invasive approaches. Patients with missing or “unknown” data regarding the following variables were excluded: ASA score, anastomotic leak, height and weight, functional health status, or preoperative ventilator dependence.

### 2.3. Exposure

The exposure of interest was the surgical approach, either LRN or RARN. We defined LRN to include “laparoscopic” and “laparoscopic with unplanned conversion to open”, and RARN to include “robotic”, “robotic with open assist (i.e., hand assist)”, and “robotic with unplanned conversion to open”.

### 2.4. Outcomes

The primary outcome comprised a composite of major complications within 30 days of surgery, including mortality, return to the operating room, myocardial infarction (MI), and CVA. The secondary outcomes included individual primary complications as well as unplanned intubation, ventilator dependence exceeding 48 h, sepsis, urinary tract infection, surgical site infection, pulmonary embolism, deep vein thrombosis, prolonged length of stay (LOS) (defined as more than 3 days, which was the median LOS in our cohort), transfusions within 72 h after surgery, wound dehiscence, unplanned readmission, acute renal failure, and *Clostridium difficile* infection. Additionally, we examined the complications specific to nephrectomy, including the development of lymphocele or other lymphatic leaks, unplanned conversion to open, or prolonged NPO or NGT use.

### 2.5. Covariates

Our analysis incorporated demographic and clinical characteristics such as age, sex, body mass index (BMI), smoking status, ASA score, chronic steroid use, a modified frailty index, current dialysis status, lymph node dissection during operation, and pathologic stage. These covariates were selected based on their clinical relevance to postoperative outcomes and their potential to influence the choice of surgical approach and postoperative results.

### 2.6. Statistical Analysis

The data were presented as mean values with standard deviations (SD) for continuous variables, the median and interquartile range (IQR) for skewed continuous variables, and frequency for categorical variables. An absolute standardized mean difference (SMD) value < 0.1 was used as the cutoff for sufficient balance. Propensity score matching (PSM) was used to minimize inherent differences between patients who underwent LRN or RARN. Propensity scores were calculated using a multivariable logistic regression model involving the surgical approach as the dependent variable and the listed covariates. We performed 1:1 nearest neighbor matching using a caliper width of 0.009, which was defined as having a maximum width of 0.18 standard deviation of the logit of the estimated propensity score [17].

We utilized conditional logistic or linear regression to assess the association between the surgical approach and our outcomes of interest to account for the clustering created by matching. Interaction terms between the surgical approach and predefined subgroups, such as age, BMI, and pathologic T stage, were included to assess the heterogeneity of effects.

All data management and statistical analyses were conducted using STATA version 17 (StataCorp. College Station, TX, USA, 2021). We considered a two-tailed *p*-value threshold of 0.05 for statistical significance for all comparisons.

## 3. Results

### 3.1. Baseline Demographics

Our cohort comprised 1545 patients (mean age: 62.97 ± 11.97 years) undergoing RN between 2019 and 2021, including 722 (46.7%) RARN and 823 (53.3%) LRN. Before PSM, we found statistically significant differences in ASA score: patients with an ASA score of 1–2 were more likely to undergo RARN. The PSM resulted in well-balanced covariates (as listed in Table 1) between groups (standardized mean difference < 0.1, Table 1) with 672 patients in each group. After PSM, 712 (52.8%) patients had T1-stage, 166 (12.35%) had T2-stage, and 466 (34.67%) had T3-stage kidney cancer.

### 3.2. Primary Outcomes

We did not identify a difference between LRN and RARN with respect to the composite outcome of major complications (OR 0.93, 95% CI: 0.43, 2.00, *p* = 0.848) (Table 2).

### 3.3. Secondary Outcomes

We did not observe a difference between RARN and LRN in any individual components of the primary outcome (30-day mortality, return to the operation room, cardiac arrest, MI, or CVA). The patients receiving LRN had a higher SSI (2.68%) relative to RARN (1.19%) (OR 2.28, 95% CI: 1.01, 5.16, *p* = 0.047). Furthermore, 18.6% of the patients undergoing LRN had a prolonged LOS (defined as LOS > 3 days, which was the median LOS in the cohort) compared with 12.95% of RARN patients (OR 1.54, 95% CI: 1.15, 2.06, *p* = 0.004) (Table 2).

### 3.4. Nephrectomy-Specific Outcomes

The total operative time for the patients who underwent RARN was 30.67 min longer than those who underwent LRN (estimated coefficient: 30.67, 95% CI: 23.15, 38.18 min, *p* < 0.001) (Table 2). However, the patients receiving RARN had significantly lower rates of conversion to open RN (1.16%) compared with those who underwent LRN (4.3%) (OR 3.70, 95% CI: 3.25, 4.15, *p* < 0.001). We did not find statistically significant evidence to support the difference in the development of lymphocele or other lymphatic leaks, prolonged postoperative NPO, or NGT use between the LRN and RARN groups (Table 2).

### 3.5. Subgroup Analysis

**Figure 1:**
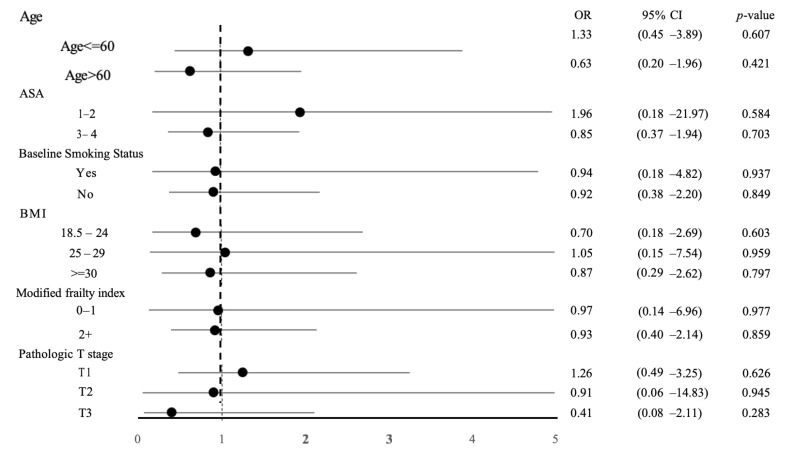
Forrest plot depicting the association between operative modality (RARN vs. LRN) and major complications among subgroups after PSM. OR, odds ratio; CI, confidence interval; PSM, propensity score matching; RARN, robotic-assisted radical nephrectomy; LRN, laparoscopic radical nephrectomy.

**Figure 2:**
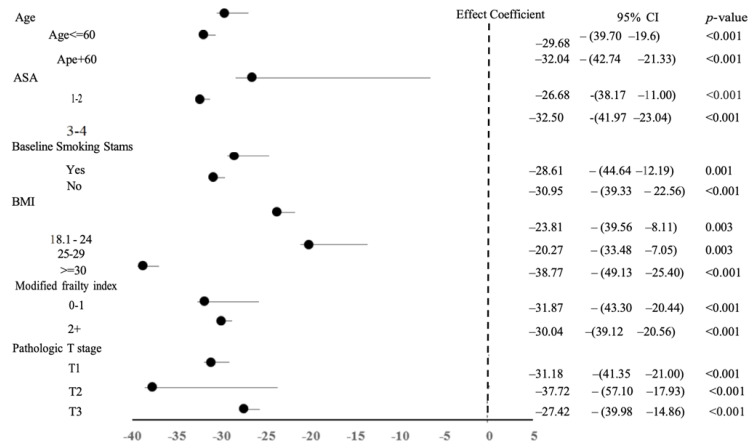
Forrest plot depicting the association between operative approach (RARN vs. LRN) and total operation time among subgroups after PSM. OR, odds ratio; CI, confidence interval; PSM, propensity score matching; RARN, robotic-assisted radical nephrectomy; LRN, laparoscopic radical nephrectomy.

We examined the combined composite outcomes of major complication results within predefined subgroups to explore the variations in how the chosen operative method influences the primary outcome. Our analysis found no heterogeneity in the operative modality and the primary outcome across the studied subgroups (Figure 1). Notably, there was an increased difference in total operative time between the RARN and LRN groups when patients were older, had a higher ASA score, had a higher BMI, and had a pathologic T2 tumor stage (Figure 2).

## 4. Discussion

We utilized the NSQIP Targeted Nephrectomy dataset to examine the granular, 30-day complications among patients undergoing robotic or laparascopic nephrectomy for kidney cancer in the United States between 2019 and 2021. The patients treated by a robotic approach (RARN) had comparable rates of major perioperative surgical complications but a lower likelihood of conversion to open surgery and surgical site infection (SSI), at the expense of longer operative time in comparison to a laparoscopic approach (LRN).

Contextualizing these findings with prior work requires an understanding of the treatment landscape. It is important to note that our study took a look at contemporary findings while previous studies were limited in scope, focusing on early-stage kidney cancer, including mostly elderly patients over the age of 75, or were conducted before 2018. As a result, their findings may not be generalizable to a broader population [7,8,10,15]. In 2015, RARN made up only 27.0% of all RNs, while in our study (range: from 2019 to 2021), we found that RARN comprised 53.3% of RNs amongst the 700 hospitals participating in NSQIP [15]. Although the prevalence of RARN has increased, our findings are broadly consistent with earlier studies. A meta-analysis involving 52,799 patients diagnosed before 2015 found that patients with renal mass who underwent LRN or RARN had similar overall major, intraoperative, and postoperative complications [18]. In this contemporary cohort, we demonstrated that RARN has similar composite major complications relative to LRN among kidney cancer patients. In contrast to our findings, Gershman et al. observed that RARN was associated with lower rates of intraoperative and postoperative complications versus LRN in a database study from 2010 to 2013 [19]. These divergent findings may be attributed to the fact that, at the time, robotic cases were less common than now, and patients were specifically optimized for robotic cases. However, with more training and the increased surgical dexterity offered with the robotic approach, robotic surgery is now more widespread and is more likely a substitute for open surgery than LRN. Therefore, patients are not necessarily selected, and in fact, technically complex cases are chosen for RARN which is perhaps the reason we see a similarity in postsurgical outcomes between LRN and RARN [20].

We observed a shorter LOS among patients who underwent RARN than LRN. Others have found the same effect [18,21,22,23] or observed that patients undergoing RARN or LRN have similar LOS [9,15]. This difference may exist for various reasons including inconsistent definitions of prolonged LOS and heterogeneity within patient populations. Furthermore, our findings indicate that RARN was associated with fewer SSIs than LRN, the mechanism of which is unclear. However, this finding may be due to the fact that laparoscopic surgery inherently has more “hand-assisted” time. This relative increase in the duration of port manipulation may increase the potential for infection versus the extraction excision that is needed in RARN.

Operative times have drastically decreased for both RARN and LRN over time, with the latter consistently being found to be shorter. The mean operative time for RARN was in the 220–350 min range for RARN while LRN was in the 175–265 min range in the early to mid-2000s [24,25]. More consistent with our study, [18] Crocerossa et al., examining studies from 2006 to 2019, found RARN to have a mean time of 177 min and LRN to have a mean time of 146 min. The reduction in duration for RARN and LRN over time indicates accumulated experience and progression along learning curves [26]. However, the persistent longer operation time in RARN likely reflects the inherent time needed for robot docking and undocking.

We found that RARN was associated with a lower risk of unplanned conversion to open surgery. In contrast, a higher conversion rate to an open surgical approach was observed in a study involving 319 RN cases between 2010 and 2014 [10]. This difference may be explained by the fact that as RARN became more widely adopted and surgeon experience has increased, the patient and tumor characteristics that necessitated the need to convert to an open surgical approach decreased [24]. Various other factors such as sample size, the surgeon’s judgment, and the diversity in patient characteristics may explain study heterogeneity.

Although we were unable to assess cost in our study due to the lack of financial information in NSQIP, the previous literature has discussed a financial cost difference between RARN and LRN: RARN has been associated with an increased overall cost [15,27]. Jeong et al. found that RARN was associated with an increased operative time and higher overall hospital costs [15]. As with our study, RARN was associated with a decreased LOS relative to LRN; however, this LOS advantage may not be sufficient to mitigate the increased costs (e.g., disposables or OR time) of robotic surgery. Yu et al. showed that RARN was again associated with a decreased LOS and resulted in fewer deaths and complications compared with open surgery, but was associated with a higher cost [27]. Given that our data do not support a clear benefit to major complications, hospitals and surgeons should consider a multitude of factors such as patient outcomes, case complexity, and affordability when selecting between LRN and RARN to optimize the value of robotic surgery. Furthermore, while robotic surgery is increasingly pervasive, it is not yet widely available worldwide owing to the lack of capital and disposable costs. In alignment with the principles of value-based care, our study re-affirms the value and affordability of laparoscopic radical nephrectomy given that the vast majority of complications examined in this study were noninferior to robotic surgery.

There are several limitations to this retrospective cohort study. First, the ACS-NSQIP dataset lacks granular information on clinical tumor staging at diagnosis, which could have facilitated a more precise balancing of the two surgical approaches. Despite employing PSM to address inherent selection bias, our model may not incorporate other unknown or poorly measured confounders such as surgeon and anesthesiologist experience, case volume, and facility resources, all of which can impact perioperative outcomes. Moreover, this analysis is constrained by the relatively recent establishment of the Targeted NSQIP database, allowing only three years of data to be examined. In addition, NSQIP data limits our analysis to 30-day perioperative outcomes; therefore, there is a need for studies examining longer-term perioperative and oncological outcomes. Lastly, our study lacks data related to surgeons and facilities. Inexperienced centers may experience extended setup times, and variability in surgeon expertise may potentially influence perioperative outcomes [26].

Our contemporary study, although conducted in the United States, analyzed data from a multitude of centers including urban, rural, academic, and community centers. Due to the gamut of patients, surgeons, anesthesiologists, and facilities, our study can be applied to a variety of settings.

## 5. Conclusions

This population-based retrospective study found comparable major complications between robotic or laparascopic radical nephrectomy for renal cell carcinoma with respect to mortality, return to the OR, cardiac arrest, or stroke. We found unique differences favoring the robotic approach over laparascopic surgery with respect to the risk of SSI, the LOS, and conversion rate to open surgery.

## Figures and Tables

**Table 1:** Baseline characteristics before and after propensity score matching among kidney cancer patients undergoing RARN or LRN.

Variables	Before PSM				After PSM			
	Total	RARN	LRN	SMD	Total	RARN	LRN	SMD
	*n* = 1545	*n* = 722	*n* = 823		*n* = 1344	*n* = 672	*n* = 672	
Age	62.97 ± 11.97	62.47 ± 11.97	63.41 ± 11.96	−0.079	62.93 ± 11.82	62.98 ± 11.70	62.89 ± 11.95	0.007
Sex								
Female	519 (33.59)	233 (32.27)	286 (34.75)	0.053	455 (33.85)	225 (33.48)	230 (34.23)	0.016
Male	1026 (66.41)	489 (67.73)	537 (65.25)		889 (66.15)	447 (66.52)	442 (65.77)	
BMI	30.90 ± 7.03	30.75 ± 6.80	31.03 ± 7.23	−0.039	30.96 ± 6.99	30.96 ± 6.90	30.95 ± 7.09	0.001
Current smoking status								
No	1294 (83.75)	605 (83.80)	689 (83.72)	0.002	1127 (83.85)	567 (84.38)	560 (83.33)	0.028
Yes	251 (16.25)	117 (16.20)	134 (16.28)		217 (16.15)	105 (15.63)	112 (16.67)	
ASA								
1–2	498 (32.23)	255 (35.32)	243 (29.53)	0.124	447 (33.26)	221 (32.89)	226 (33.63)	0.016
3–4	1047 (67.77)	467 (64.68)	580 (70.47)		897 (66.74)	451 (67.11)	446 (66.37)	
Baseline dialysis status								
No	1456 (94.24)	674 (93.35)	782 (95.02)	0.71	1267 (94.27)	634 (94.35)	633 (94.20)	0.006
Yes	89 (5.76)	48 (6.65)	41 (4.98)		77 (5.73)	38 (5.65)	39 (5.80)	
Chronic steroid use								
No	1467 (94.95)	684 (94.74)	783 (95.14)	0.018	1276 (94.94)	638 (94.94)	638 (94.94)	<0.001
Yes	78 (5.05)	38 (5.26)	40 (4.86)		68 (5.06)	34 (5.06)	34 (5.06)	
Modified frailty index								
Score 0–1	479 (31.00)	226 (31.30)	253 (30.74)	0.012	424 (31.55)	209 (31.10)	215 (31.99)	0.019
Score 2+	1066 (69.00)	496 (68.70)	570 (69.26)		920 (68.45)	463 (68.90)	457 (68.01)	
Lymph node dissection status								
No	1318 (85.31)	609 (84.35)	709 (86.15)	0.051	1152 (85.71)	574 (85.42)	578 (86.01)	0.017
Yes	227 (14.69)	113 (15.65)	114 (13.85)		192 (14.29)	98 (14.58)	94 (13.99)	
Pathologic T stage								
T1	799 (52.46)	371 (52.40)	428 (52.52)	0.066	712 (52.98)	357 (53.13)	355 (52.83)	0.037
T2	188 (12.34)	80 (11.30)	108 (13.25)		166 (12.35)	79 (11.76)	87 (12.95)	
T3	536 (35.19)	257 (36.30)	279 (34.23)		466 (34.67)	236 (35.12)	230 (34.23)	

Notes: 1. Data are presented as mean ± SD, median (IQR) for continuous measures, and *n* (%) for categorical measures. 2. Propensity score matching (PSM) with a 1:1 ratio between robotic and laparoscopic operative approach was performed through a greedy algorithm based on a caliper of 0.009, equivalent to 0.18 standard deviation (SD) of the logit of the estimated propensity score, 16. A logistic regression model was fitted to the operative approach as a binary dependent variable, while covariates included all variables in the table. 3. BMI, Body Mass Index; ASA, American Society of Anesthesiologists physical status score; PSM, propensity score matching; RARN, robotic-assisted radical nephrectomy; LRN, laparoscopic radical nephrectomy; SMD, standardized mean difference. An absolute SMD value < 0.1 was used as the cutoff for sufficient balance.

**Table 2:** Comparison of common surgical complications between RARN or LRN among kidney cancer patients after PSM.

Variables	Total	RARN	LRN	LRN vs. RARN	*p*
	*n* = 1344	*n* = 672	*n* = 672	OR (95% CI)	
Major Complications	27 (2.01)	14 (2.08)	13 (1.93)	0.93 (0.43, 2.00)	0.848
30-day Mortality	4 (0.30)	3 (0.45)	1 (0.15)	0.33 (0.03, 3.22)	0.342
Return to Operation Room	18 (1.34)	10 (1.49)	8 (1.19)	0.80 (0.31, 2.05)	0.638
Cardiac Arrest	3 (0.22)	2 (0.30)	1 (0.15)	0.50 (0.04, 5.54)	0.572
Myocardial Infarction	9 (0.67)	3 (0.45)	6 (0.89)	2.01 (0.50, 8.11)	0.327
Stroke/Cerebrovascular Accident	0 (0.00)	0 (0.00)	0 (0.00)	NA	-
Secondary Outcomes					
Unplanned Intubation	5 (0.37)	1 (0.15)	4 (0.60)	4.02 (0.45, 36.20)	0.215
Ventilator > 48 h	4 (0.30)	1 (0.15)	3 (0.45)	3.01 (0.31, 29.12)	0.342
Sepsis	5 (0.37)	2 (0.30)	3 (0.45)	1.50 (0.25, 9.06)	0.657
Urinary Tract Infection	12 (0.89)	5 (0.74)	7 (1.04)	1.40 (0.44, 4.47)	0.566
Surgical Site Infection	26 (1.93)	8 (1.19)	18 (2.68)	2.28 (1.01, 5.16)	**0.047**
Superficial Surgical Site Infection	19 (1.41)	8 (1.19)	11 (1.64)	1.38 (0.58, 3.31)	0.469
Deep Surgical Site Infection	3 (0.22)	0 (0.00)	3 (0.45)	NA	-
Organ Site Surgical Site Infection	4 (0.30)	0 (0.00)	4 (0.60)	NA	-
Pulmonary Embolism	5 (0.37)	2 (0.30)	3 (0.45)	1.50 (0.25, 9.06)	0.657
Deep Vein Thrombosis	6 (0.45)	1 (0.15)	5 (0.74)	5.03 (0.58, 43.35)	0.142
Prolonged Length of Stay	212 (15.77)	87 (12.95)	125 (18.60)	1.54 (1.15, 2.06)	**0.004**
Transfusion within 72 h of Surgery	44 (3.27)	20 (2.98)	24 (3.57)	1.21 (0.65, 2.23)	0.547
Hospitalized > 30 days	14 (1.04)	6 (0.89)	8 (1.19)	1.34 (0.46, 3.90)	0.594
Wound Disruption	6 (0.45)	4 (0.60)	2 (0.30)	0.50 (0.09, 2.74)	0.424
Unplanned Readmission	47 (3.50)	19 (2.83)	28 (4.17)	1.49 (0.84, 2.66)	0.173
Acute Renal Failure	3 (0.22)	2 (0.30)	1 (0.15)	0.50 (0.04, 5.54)	0.572
Clostridium Difficile	3 (0.22)	2 (0.30)	1 (0.15)	0.50 (0.04, 5.54)	0.572
Nephrectomy-Specific Outcomes					
Lymphocele or Other Lymphatic Leaks	14 (1.04)	8 (1.19)	6 (0.89)	0.75 (0.26, 2.18)	0.594
Prolonged Postop NPO or NGT use	20 (1.49)	8 (1.19)	12 (1.79)	1.51 (0.61, 3.74)	0.375
Total Operation Time	162 ± 69	177 ± 72	146 ± 63	−30.67 (−38.18, −23.15) *	**<0.001**
Unplanned Conversion Rate to Open Approach	37(2.75)	8 (1.16)	29 (4.30)	3.70 (3.25, 4.15)	**<0.001**

Notes: * Estimated coefficient and 95% CI. 1. Conditional logistic regression was used to account for the clustering created by matching. 2. Prolonged LOS was defined as LOS > 3 days, which was the median LOS in our cohort. 3. Prolonged postoperative NPO or NGT was defined if patients had prolonged postoperative NPO or NGT use. 4. The bold *p*-values indicate statistical significance. 5. NPO, postoperative nil per os; NGT, nasogastric tube; OR, odds ratio; CI, confidence interval; PSM, propensity score matching; RARN, robotic-assisted radical nephrectomy; LRN, laparoscopic radical nephrectomy.

## Data Availability

This data is publicly available through the National Surgical Quality Improvement Program.
